# Antimalarial Drug Combination Predictions Using the Machine Learning Synergy Predictor (MLSyPred©) tool

**DOI:** 10.1007/s11686-023-00765-z

**Published:** 2024-01-02

**Authors:** Abiel Roche-Lima, Angélica M. Rosado-Quiñones, Roberto A. Feliu-Maldonado, María Del Mar Figueroa-Gispert, Jennifer Díaz-Rivera, Roberto G. Díaz-González, Kelvin Carrasquillo-Carrion, Brenda G. Nieves, Emilee E. Colón-Lorenzo, Adelfa E. Serrano

**Affiliations:** 1 Center for Collaborative Research in Health Disparities, University of Puerto Rico, Medical Sciences Campus, San Juan, PR, USA; 2 Department of Microbiology and Medical Zoology, School of Medicine, University of Puerto Rico, Medical Sciences Campus, San Juan, PR, USA

**Keywords:** Machine learning, Drug combination prediction, Drug synergy, *Plasmodium*, Malaria

## Abstract

**Purpose:**

Antimalarial drug resistance is a global public health problem that leads to treatment failure. Synergistic drug combinations can improve treatment outcomes and delay the development of drug resistance. Here, we describe the implementation of a freely available computational tool, Machine Learning Synergy Predictor (MLSyPred©), to predict potential synergy in antimalarial drug combinations.

**Methods:**

The MLSyPred© synergy prediction method extracts molecular fingerprints from the drugs’ biochemical structures to use as features and also cleans and prepares the raw data. Five machine learning algorithms (Logistic Regression, Random Forest, Support vector machine, Ada Boost, and Gradient Boost) were implemented to build prediction models. Implementation and application of the MLSyPred© tool were tested using datasets from 1540 combinations of 79 drugs and compounds biologically evaluated in pairs for three strains of *Plasmodium falciparum* (3D7, HB3, and Dd2).

**Results:**

The best prediction models were obtained using Logistic Regression for antimalarials with the strains Dd2 and HB3 (0.81 and 0.70 AUC, respectively) and Random Forest for antimalarials with 3D7 (0.69 AUC). The MLSyPred© tool yielded 45% precision for synergistically predicted antimalarial drug combinations that were annotated and biologically validated, thus confirming the functionality and applicability of the tool.

**Conclusion:**

The MLSyPred© tool is freely available and represents a promising strategy for discovering potential synergistic drug combinations for further development as novel antimalarial therapies.

## Introduction

Malaria is considered the most devastating parasitic disease in the world. Antimalarial drug resistance is a global public health problem that is responsible for the failures of both individual malaria treatment and global malaria control. The loss of sensitivity to commonly used drugs has resulted in an alarming increase in morbidity and mortality [1]. Resistance to first-line antimalarial drugs such as chloroquine, other quinolines, and artemisinin has caused increasing morbidity and mortality in children worldwide [2]. A common and fundamental strategy to overcome drug resistance is the use of drug combination therapies, such as artemisinin combination therapies (ACTs), to slow the development of drug resistance [2].

The increasing burden of multidrug-resistant organisms is a reality and a global problem, not only for malaria but also for multiple microbes. Alarmingly, antimicrobial resistance in bacteria, parasites, viruses, and fungi infections is one of the most severe global public health threats, resulting in increasing cases and deaths, and is predicted to cause 10 million deaths worldwide per year by 2050 [3]. As an example, tuberculosis is projected to become the leading cause of death worldwide due to the increasing drug resistance of *Mycobacterium tuberculosis* to approved treatments [4]. The MLSyPred© tool described here can be used to predict synergistic combinations to improve treatment and prevent the development of drug resistance in these diseases.

Drug combinations offer a promising strategy to extend the useful lives of drug components and tackle the emergence of drug resistance. More importantly, drug combinations can exhibit synergism, where the efficacy of the combination is greater than the sum of the effectiveness of individual drugs [5–8]. Computational methods can predict synergistic drug combinations prior to laborious and expensive wet lab validation with the candidate drugs. There are two main types of computational methods for predicting synergistic drug combinations: mechanistic methods and machine learning (ML) models [5, 9–18]. Mechanistic methods are based on biological processes centered on the input-output relationship. In contrast, ML-based methods use algorithmic approaches to categorize data or predict an outcome. Both computational methods can use diverse datasets, such as chemical structures, biological network interactions, and omics data. An example of a mechanistic method includes the Ranking-system of Anti-Cancer Synergy (RACS), which ranks cancer-related drug pairs on drug targeting networks and transcriptomic profiles [19]. The Drug-induced genomic residual effect (DIGRE) is a mechanistic tool that creates models based on drug response curves and gene expression changes after drug treatment to predict drug combination effects [14]. DrugComboRanker is another tool that ranks potential synergistic drug combinations based on genomic information using a Bayesian non-negative matrix factorization approach. Some examples of ML-based methods include DeepSynergy, a deep learning approach to predict synergy using chemical fingerprints of the drugs and gene expression profiles of a cell line of interest as an input [20]. In addition, Cuvitoglu *et al*. (2019) developed an ML classification model to predict drug synergy pairs using transcriptomic data from cancer cells and biological network analysis [21]. Meanwhile, others, such as DrugComb [22] and the Probability Ensemble Approach [23], predict synergism using the structural composition of chemical compounds. However, many available tools have only been tested in specific cell lines, types of cancer, or specific diseases [24].

Despite the availability of several computational methods for predicting drug synergy, there is currently no flexible tool that allows the integration of different data types and methods to create synergistic predictive models. Mason *et al*. 2017 and Mason *et al*. 2018 described Combinations synergy estimation (CoSynE), which is a ML method that uses compound chemical structures and experimental combination screening data to predict synergistic drug pairs [25, 26]. The CoSynE tool has been used to predict antimalarial and antibiotic drug combinations, but is not widely available [25, 26]. Due to the limitation of accessing CoSynE, we implemented the freely accessible MLSyPred© tool to predict antimalarial drug combinations.

Herein, this paper describes MLSyPred©, a data sciencebased tool that creates ML-based models to successfully predict antimalarial drug combinations to be further evaluated as treatments. This tool is based on the five essential phases of a data science life cycle, namely, (1) Data Understanding/Data Pre processing; (2) Data Wrangling; (3) Model Planning; (4) Model Building/Modeling; and (5) Results.

## Materials and Methods

### Datasets of Drug Combinations

Three biologically validated datasets of antimalarial strains of *Plasmodium falciparum* (3D7, Dd2, and HB3) [26] were used to test the implementation and application of the MLSyPred© tool. An antibiotic dataset was tested to validate the functionality and applicability of this tool [25]. All datasets were derived from the NIH National Center for Advancing Translational Sciences public domain resources and downloaded for this study. Each dataset was divided into training and validation datasets.

The antimalarial dataset included drugs and compounds that were biologically evaluated by Mott *et al*. [27] and tested by Mason *et al*. [26] for three different strains of *P. falciparum* (3D7, HB3, and Dd2). The dataset contained 79 antimalarials divided into 56 for training and 23 for validation, resulting in 1540 combinations of compounds paired as a training set and 231 for the validation set. The antimalarial dataset contained drugs currently used for malaria treatment, including dihydroartemisinin, artemether, artesunate, chloroquine, mefloquine, amodiaquine, and piperaquine.

The antibiotic dataset was previously biologically tested against *Escherichia coli* [25]. The dataset contained 24 antibiotics divided into 18 for training and 6 for validation, resulting in a training set of 153 combinations and a validation set of 15 combinations. The antibiotic dataset contained drugs currently available to treat bacterial infections, including chloramphenicol, clarithromycin, erythromycin, fusidic acid, gentamicin, rifampicin, spectinomycin, and tetracycline.

### Computational Applications to Implement MLSyPred©

The MLSyPred© tool encompasses several phases to incorporate as many modules as needed to manipulate data types and computational methods. Different platforms, programming languages, and existing applications were used, such as Anaconda Navigator v1.9.12 [28], Integrated Development Environment (IDE) Spyder v4.1.5 [29], Jupyter Notebook v6.2.0 [28], and RDKit v.2021.03.1 [30]. Anaconda Navigator was used to launch Jupyter Notebook and IDE Spyder, both used in the MLSyPred© tool.

**Fig. 1:**
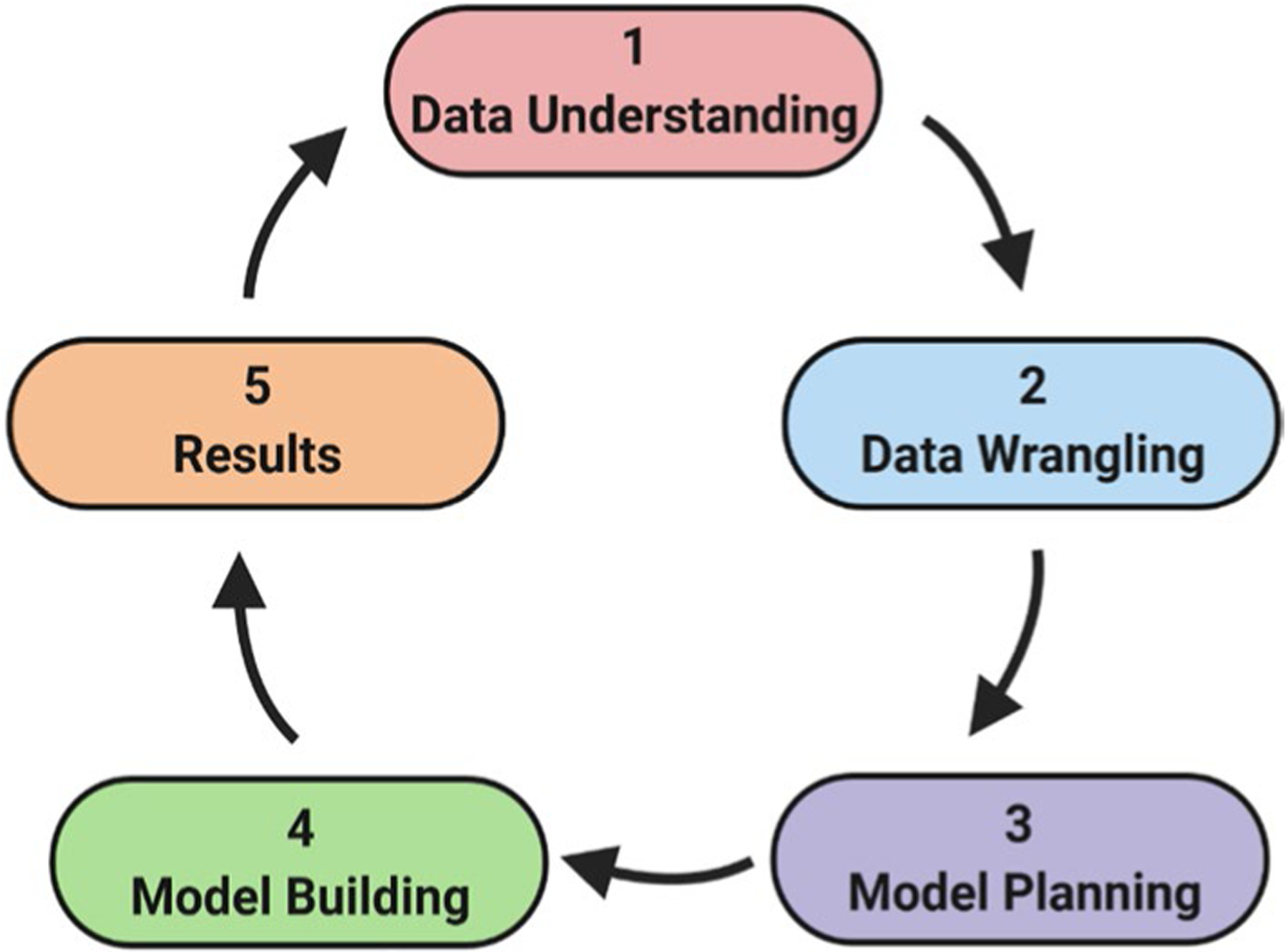
The life cycle of a Data Science Project. Image created with BioRender.com

**Fig. 2:**
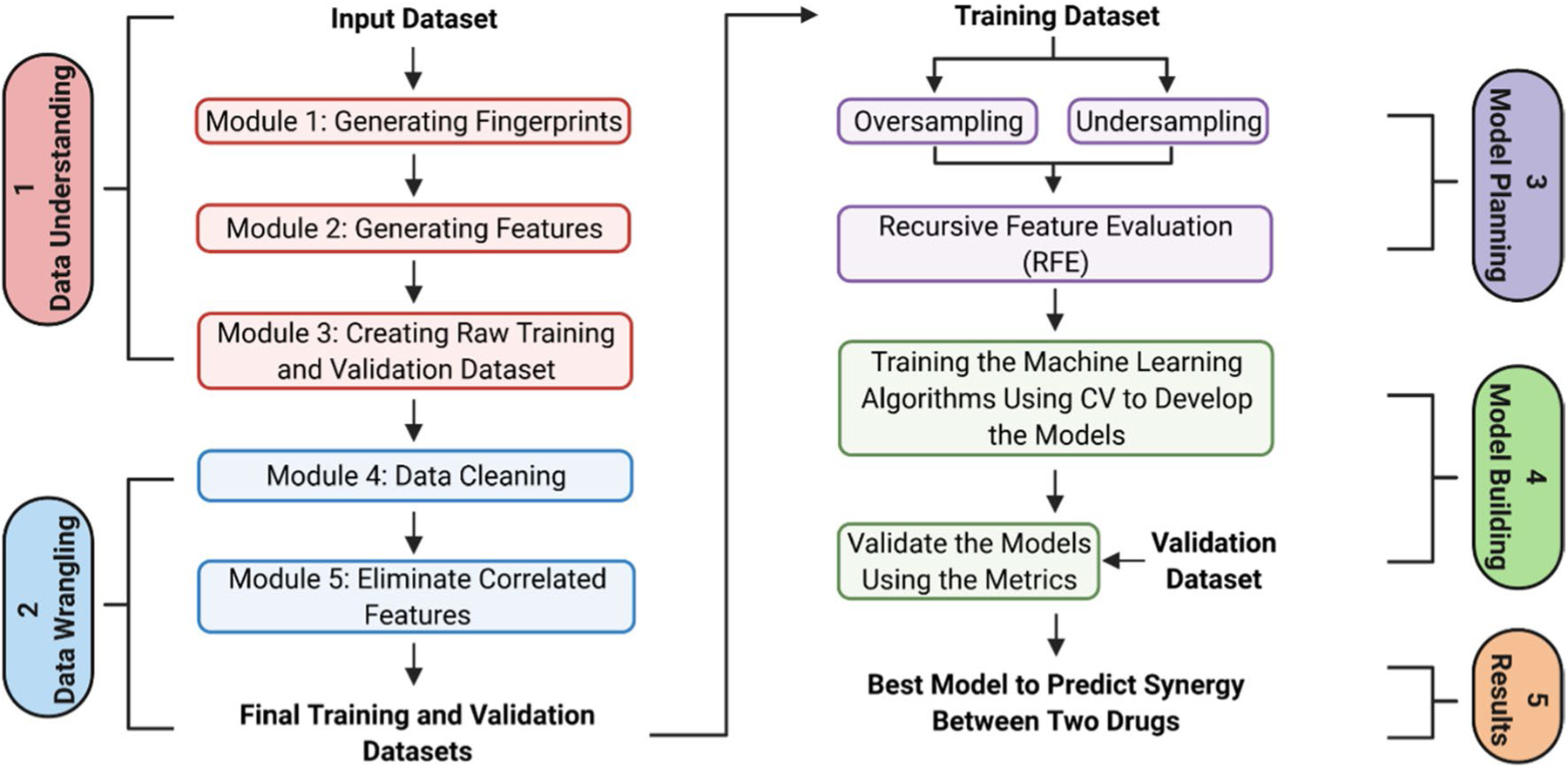
Overview of the MLSyPred© tool for each phase of the MLSyPred© tool. Image created with BioRender.com

The Jupyter Notebook was used as the baseline interface for the MLSyPred© tool to implement the five phases of the data science project, as shown in Fig. 1. This platform also allows the addition of modules according to the needs by using programming languages such as Python v3.9.1, R, Java, and C + + , among others, and provides the ability to work online or offline (Fig. 2). In addition, Spyder was used to implement different Python modules of the ML model included in the different phases of the MLSyPred© tool. MLSyPred© is flexible and accepts any module coded in any programming language.

### Implemented Modules in MLSyPred©

To test the MLSyPred© tool, we implemented several modules of the ML-based method (based on Mason *et al*. [25]) following five different phases of MLSyPred©. Figure 2 shows the modules implemented in each phase of the MLSyPred© tool. As input data, we used compound chemical structures, i.e., the Simplified Molecular input line entry system (SMILES), to extract characteristics, such as molecular fingerprints [31]. The RDKit software package v.2021.03.1 was used to calculate molecular fingerprints, MACCS keys [32], and Morgan fingerprints [33]. The MACCS keys and Morgan fingerprint methods generated molecular fingerprints for atom membership. MACCS keys were calculated with a default key size parameter of 166 and Morgan fingerprints with 1024-bit and 2048-bit vectors.

An ML-based method (as described by Mason *et al*. [25]) was implemented to include the MLSyPred© tool to create the predictive models. We used the Scikit-learn library v0.24 [34] to implement ML algorithms. We executed the following ML algorithms: Random Forest (RF) [35]; Logistics Regression (LR) [36]; Support vector machine (SVM) [37]; Gradient boost (GB) [38]; and AdaBoost (AB) [38].

After training the ML algorithms, different metric evaluations were performed to determine the most precise model. First, the confusion matrix [39] was calculated and then the metrics: accuracy, precision, recall, F1, and, most importantly, the AUC ROC (area under receiver operating characteristics) scores [40]. The AUC ROC metric was used to identify the best model; the highest AUC ROC score was the best predictive model for distinguishing between paired compounds.

### Evaluation Metrics

Each model was evaluated using the following machine learning metrics: AUC ROC, precision, accuracy, recall scores, and F1 scores. These metrics were calculated from balanced weights for the antibiotic and Dd2 antimalarial datasets and unbalanced weights for the 3D7 and HB3 antimalarial datasets. Then, the optimal number of features was computed for each dataset, and with these calculations, the ML model algorithms were run to select the best-performing model for each dataset based on the AUC ROC scores. Subsequently, the best-performing model parameters for each dataset were identified and annotated. Precision scores indicate that positive class predictions are genuinely positive, whereas recall quantifies positive class predictions from the total positive classes from the dataset. In summary, F1 scores balance both precision and recall scores. High precision and recall scores signify that the best-performing model yields all the correctly predicted positive results. Accuracy scores indicate the ratio of correctly predicted classes to total observations in symmetrical datasets; therefore, high accuracy is expected to assess the positive predictions of the model. Evaluation metrics for the three antimalarial strain ML-based models are listed in Table 1. In addition, the sampling method was considered, and the sampling process was repeated 1000 times to prevent data bias, and an average was taken as the result.

### Validation of Predicted Drug Combinations from Existing Drug Combinations

To evaluate and validate the MLSyPred© tool, 23 compounds were selected for pairwise validation, and 253 combinations were evaluated using the strain MLSyPred©-MalDd2 and the model with the best AUC ROC score, Dd2. The validated drug combinations (9) predicted to generate synergistic combinations are listed in Table 2. The synergistic combinations obtained using the MLSyPred© tool were validated using a dataset from Kalantar-Motamendi *et al*. [41].

## Results

### MLSyPred© Phase 1—Data Understanding/Preprocessing

Phase 1 consisted of three modules to identify information and implement Data Preprocessing to create the training and validation datasets.

#### Module 1: Generating Fingerprints

**Fig. 3:**
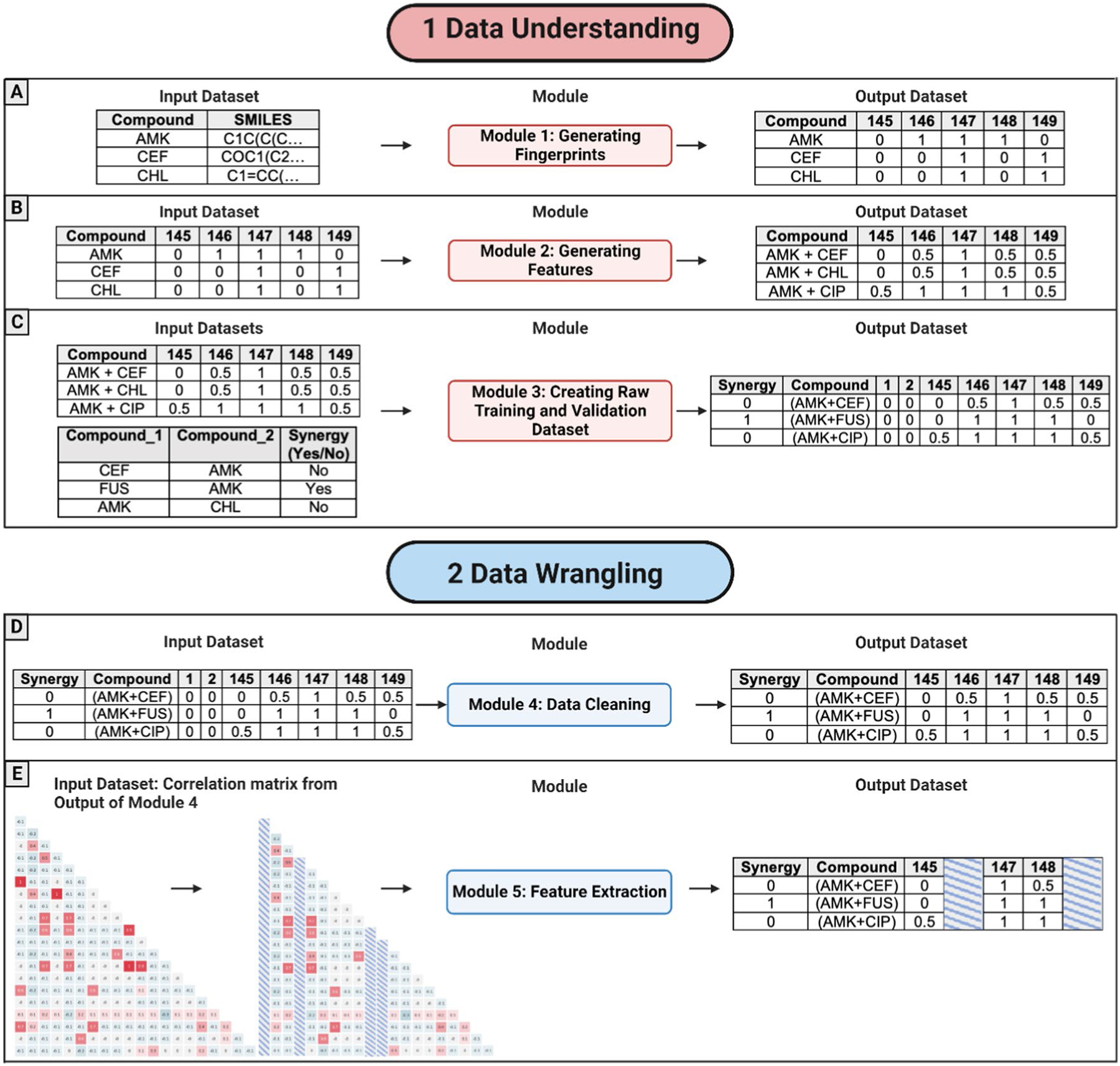
Representations of the input and output datasets for each module in the Data Understanding and Data Wrangling phases. Image created with BioRender.com

This module was created to generate bit vectors for each drug individually by computing the MACCS key and/or Morgan fingerprints (Fig. 3A). The input data were drug names and their SMILES representations (Fig. 3A—input). Scripts were implemented to compute: (1) 166-MACCS key fingerprints, (2) 1024 Morgan fingerprints, and (3) 2048-Morgan fingerprints. To create bit vectors, the MACCS key or Morgan fingerprints are assigned ‘1’ when a given substructure is present in the drug or ‘0’ if the substructure is absent (Fig. 3A—output).

#### Module 2: Generating Features

The output data were the drug name and a bit vector for each drug corresponding to the fingerprints (Fig. 3B—output). This module was designed for two general tasks:

1. generate pairwise drug combinations using all drugs, and
2. for each pairwise drug combination (e.g., *Drug*_1_ with *Drug*_2_), compute the average of the fingerprints (*I* fingerprints) that correspond to each compound as:
  ∑_*i*_(*Drug*_1_ [*i], Drug*_2_ */*2 where
  *i* = 1..166 for MACCS keys fingerprints or
  *i* = 1..1024 for 1024 Morgan fingerprints or
  *i* = 1..2048 for 2048-Morgan fingerprints.

The input file included drug identification and the bit vector that represented the absence (0) or presence (1) of the substructure in the compound (Fig. 3B—input). The output file (Fig. 3B—output) included the drug combination ID (e.g., Drug_1__Drug_2_) along with the computed feature values as follows:

0.5 if one of the two drugs included fingerprints equal to 1, i.e., {*Drug*_1_ = 1, *Drug*_*2*_ = 0*orDrug*_*1*_ = 0, *Drug*_*2*_ = 1.
0 if the two drugs included fingerprints equal to 0, i.e., {*Drug*_1_ = 0, *Drug*_2_ = 0.
1 if the two drugs included fingerprints equal to 1, i.e., {*Drug*_1_ = 1, *Drug*_2_ = 1

#### Module 3: Creating Raw Training and Validation Dataset

Once all the features were generated, the training and validation datasets were created using Module 3. The script input files were as follows: a) the output of module 2 and b) an external file with the labels (Fig. 3C—input). The second file was included per line: (a) the ID of pairwise drug combinations and (b) their pharmacodynamic activity, that is, ‘YES’ if there was synergism between these two drugs and ‘NO’ otherwise. The output of this module (Fig. 3C—output) resulted from merging the features (fingerprints) previously created for the training validation sets with the existing labels from the external file.

### MLSyPred© Phase 2—Data Wrangling

Phase 2 consisted of two modules to convert the data into an acceptable format for further analysis.

#### Module 4: Data Cleaning

This module was created to clean the raw training and validation datasets. First, the features (file columns) with the same values for all drug combinations were removed from the training set. For example, this column was deleted if an X feature had values equal to “0” or “1” for all drug combinations. After the features (file columns) from the training dataset, the same characteristics (columns) were removed from the validation set. Figure 3D—input represents an example of the input format data for this script. The output file contained data without meaningful features (columns), as shown in Fig. 3D—output.

#### Module 5: Eliminate Correlated Features

This module was designed to extract highly correlated features that may cause overfitting (an overview of this module is shown in Fig. 3E). A clean training dataset was used as the input (an example is shown in Fig. 3D—output). A Pearson correlation method (implemented as a Python function was used to compute the feature correlation matrix. This function was defined with the default parameter ‘Pearson’ to calculate a correlation index (*Ci*) for all pairwise characteristics (or columns). The correlation matrix indices had values between 0 and 1. A threshold of 0.8 was used, which means:

- if *Ci* ≥ 0.8, these two characteristics are directly correlated.
- if −0.8 < *Ci* < 0.8, these two characteristics are not correlated.
- if *Ci* ≤ −0.8, these two characteristics are inversely correlated.

Subsequently, all the direct or inversely correlated pairs were removed from the training and validation datasets.

### MLSyPred© Phase 3—Model Planning

#### Oversampling/Undersampling

Two different sampling methods, oversampling and undersampling, were implemented to manage unbalanced data. Imbalanced data usually refer to a classification problem in which the number of observations per label or class is not evenly distributed [42]. In this case, there were many data/observations for one label or class (referred to as the *majority label or class*) and fewer observations for one or more other labels or classes (referred to as the *minority labels or classes*). In this module, we implemented the synthetic minority over-sampling technique (SMOTE) [43] for over-sampling and random undersampling (RUS) [44] for undersampling. In addition, a *class weight* technique was applied in this module to balance the data using the Scikit Learn library [34].

#### Recursive Feature Evaluation (RFE)

Recursive feature selection involves selecting features (columns) that allow ML algorithms to obtain the highest accuracy [45]. The Scikit Learn library [34] determined the most relevant features. Five ML algorithms (RF, LR, SVM, GB, and AB) were used to select the most critical features that allowed us to obtain the best predictive models.

### MLSyPred© Phase 4—Model Building

#### Training the ML Algorithms Using CV to Develop the Models

Five ML algorithms were implemented to train the input dataset and establish the parameters using the validation dataset. Here, cross-validation (CV) was used to compare and select the best model for the predictive modeling problem. A size of k of five was used as the default.

#### Validate the Developed Models Using the Metrics

Here, the models learned from the step and validation datasets were used to compute a confusion matrix to determine the accuracy, precision, and AUC ROC scores. The best predictive model was the model with the highest ROC value. Therefore, the best model can be used to predict other synergistic drug combinations for the domain and dataset in which it was trained, such as antibiotics, antimalarials, or any other drug combination.

### MLSyPred© Tool Phase 5—Results

**Fig. 4:**
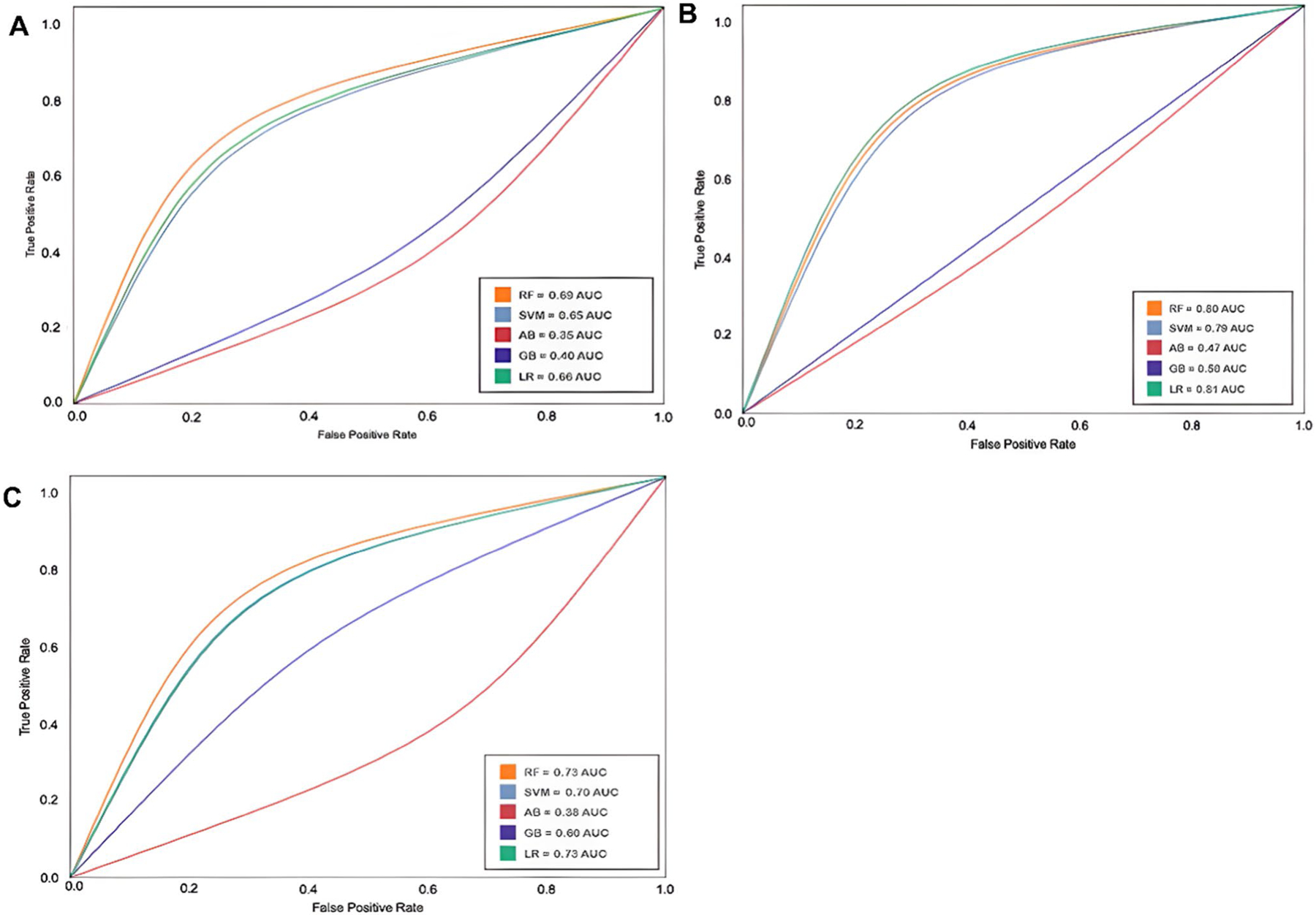
AUC ROC curves for the ML algorithms (5) trained with the antimalarial datasets. A. 3D7 strain (Random Forest 0.69), B. Dd2 strain (Logistic Regression 0.81), and C. HB3 strain (Logistic Regression 0.73). The AUC ROC curves are determined by the True Positive Rate and the False Positive Rate of each model; the higher the score, the better the model to distinguish the predictions of ‘synergy’ versus ‘no synergy’

An antimalarial drug combination validated dataset [26] of three *P. falciparum* strains [27] was used to test the MLSyPred© framework. The following fingerprints/bit vectors were calculated as characteristics for the antimalarial drug combination dataset: 1024 Morgan fingerprints and 2048-Morgan fingerprints. The selected models were obtained using 2048-Morgan fingerprints from the antimalarial dataset, as listed in Table 1. The best model was obtained to predict the synergistic drug combinations for the three strains of *P. falciparum*. The AUC ROC curves for the 3D7, HB3, and Dd2 strains are shown in Fig. 4A–C, respectively.

### Validation of Predicted Synergistic Drug Combinations Using MLSyPred©

Of the 23 compounds, 20 combinations from 12 individual compounds were predicted to have synergistic effects. These compounds were subjected to pairwise experimental validation, and 9 of 20 combinations exhibited synergy, representing 45% of the expected prediction (precision). Synergistic combinations are listed in Table 2.

### Application of a Predictive Drug Combination from Other Antimicrobial Dataset

A dataset of antibiotics tested against *E. coli* [25] was used to evaluate the application of the MLSyPred© framework to other datasets of combination antimicrobial drugs. The 166-MACCS key fingerprints were computed as the features of this dataset. The best predictive model was obtained using 166-MACCS key fingerprints from the antibiotic dataset, as shown in Table 3.

#### Best ML Predictive Model for Antibiotic Dataset

**Fig. 5:**
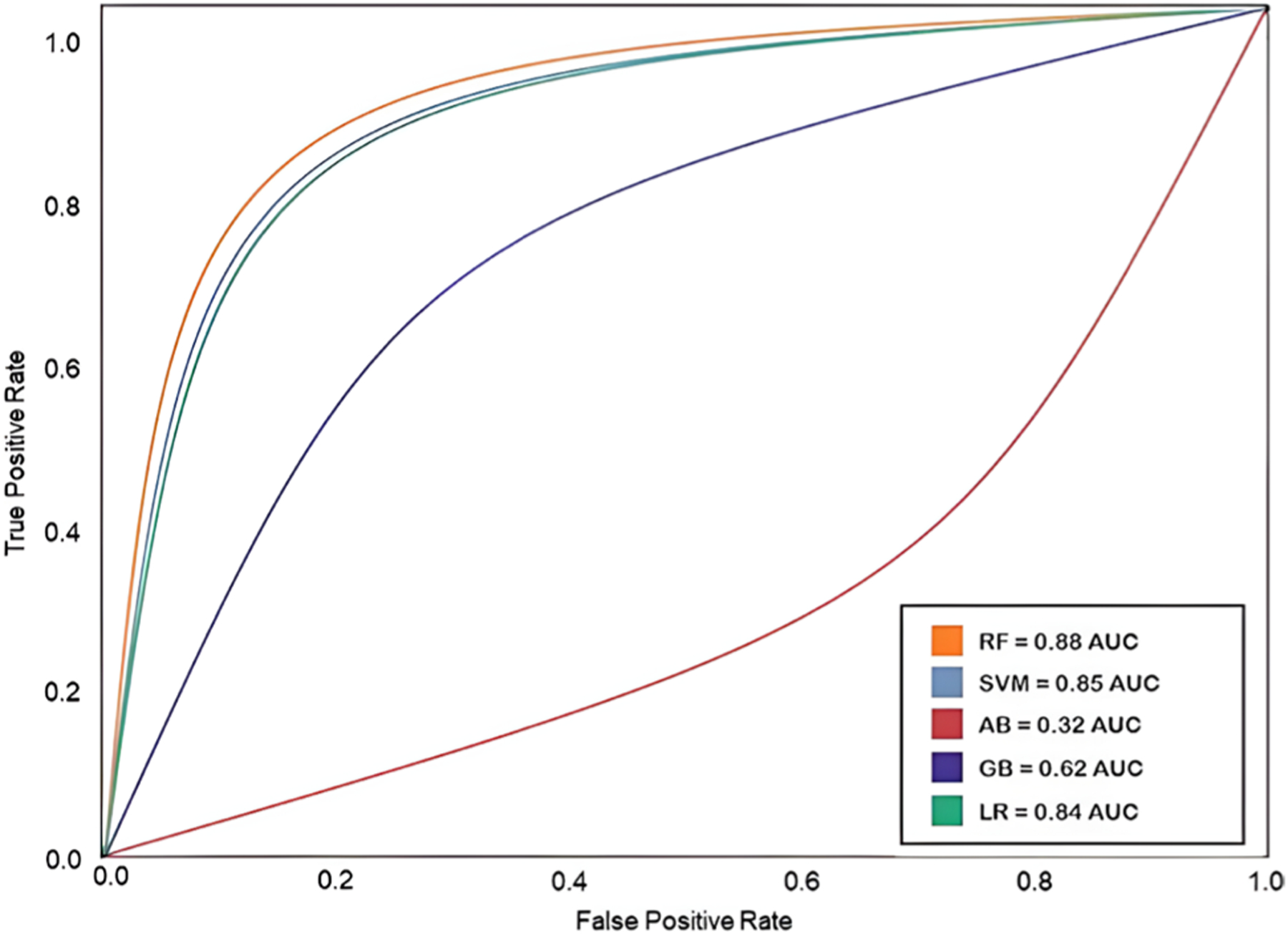
AUC ROC curves for the ML algorithms (5) trained with the antibiotic dataset. Random Forest produced the highest value of the AUC ROC score (0.88), followed by Support Vector Machine (0.85), Logistic Regression (0.84), Naive Bayes (0.62), and Ada Boost AB (0.32). The AUC ROC curves are determined by the True Positive Rate and the False Positive Rate of each model; the higher the score, the better the model to distinguish the predictions of ‘synergy’ versus ‘no synergy’

The best predictive model was obtained with the highest AUC ROC score (AUC ROC = 0.88), which was the Random Forest (Table 3). This model was named as MLSyPred©-Antb. The AUC ROC curves for the antibiotic dataset are shown in Fig. 5.

## Discussion

### Best Predictive ML Model for Antimalarial Dataset

The dataset included data from three *Plasmodium* parasite strains: 3D7, HB3, and Dd2. The best predictive models were obtained with the Logistic Regression for strains Dd2 and HB3 with AUC ROC scores of 0.81 and 0.73, respectively (Table 1). For the 3D7 strain, the best predictive model was obtained with the Random Forest, with AUC ROC scores of 0.69 (Table 1). These models were named MLSyPred©-Mal3D7, MLSyPred©-MalDd2, and MLSyPred©-MalHB3. The most relevant features for each model (40, 44, and 30, respectively) revealed the most related substructures associated with the prediction of synergism in combinatorial antimalarial therapies for each strain. Furthermore, the intersection of the most relevant characteristics for each strain could reveal the most important chemical substructures related to the available combinatorial antimalarial therapies. These results were consistent with a previous study that reported similar results for the three antimalarial datasets [26].

To evaluate the performance of the best predictive model for each antimalarial strain, we chose to highlight the precision, precision, recall, and F1 scores (Table 1). Among the three antimalarial strain datasets, the Dd2 strain showed the best parameters, since most scores generated values higher than 0.5. An accuracy score of 0.45 indicates that 45% of the predicted synergistic combinations are correct, as confirmed by the high F1 score. These results were consistent with those of a previous study that reported similar results for the three antimalarial datasets; however, some scores showed relative improvements, such as recall, accuracy, and F1 scores for the Dd2 strain [26].

### Validation of Predicted Synergistic Drug Combinations Using MLSyPred©

The existing drug combinations were corroborated by a previous study using differential gene expression data [41] (Table 2). Similarly, we identified one combination that was precisely predicted to yield synergism: apicidin and hydroxyzine. The predicted drug combinations generated from the existing drug combinations validated the MLSyPred© tool and model prediction [26]. These results were in agreement with those reported by Mason *et al*. [26]. MLSyPred© represents a promising tool for discovering potential synergistic drug combinations.

### Application of Predictive Drug Combination from Other Antimicrobial Datasets

The 48 most relevant features of MLSyPred©-Antb revealed the essential substructures that should be considered to determine the synergy in antibiotic combinatorial therapies. These results were consistent with a previous study that reported similar results for the antibiotics dataset, with an AUC ROC score of 0.88 using the Random Forest [25] (Table 3). The additional validation of the MLSyPred© tool provided by the antibiotic dataset demonstrates the flexibility and applicability of the tool, not limiting itself to the type of disease.

## Conclusion

Herein, we report the implementation, application, and availability of a freely available computational tool, MLSyPred©, built upon a data science life cycle to predict synergistic antimalarial drug combinations based on shared fingerprint features of the chemical structure of the compound. We describe the MLSyPred© tool, which incorporates methods to create predictive models for compound-drug synergistic combinations, eventually converts them into drug combinatorial therapies. The tool consists of several modules, including generating fingerprints, creating features, cleaning raw data, solving imbalanced data issues, selecting the most critical features, training and evaluating ML algorithms, and obtaining the final predictive models. To our knowledge, this tool is the first freely available tool based on a data science life cycle project that allows the incorporation of methods to create models for predict synergism in combinatorial drug therapies. The tool allows for easy reproducibility of the process to obtain the predictive models. The evaluation metrics showed promising functionality and applicability for predicting other drug combinations, with good precision (0.45) and F1 (0.59) scores. Moreover, the validation of predictive drug combinations by pairwise and expression experiments corroborated synergistic drug predictions. Drug interaction assays, such as isobologram analysis, can validate the predicted combinations to verify precision and accuracy, thus increasing and improving the tool for user requests. The MLSyPred© tool, as presented here, can expand the prediction of synergistic drug combinations to new antimalarial compounds and drugs as a mechanism to combat the drug resistance worldwide. Furthermore, this tool can be applied to other diseases and conditions by priming annotated and consistent data in a fully open-source program. The MLSy-Pred© tool is a valuable contribution to predicting effective drug combinations for multiple diseases, freely available to researchers and scientists worldwide.

## Figures and Tables

**Table 1:** The evaluation metrics for the best predictive ML model of the antimalarial dataset per strain, including the dataset, the ML algorithm, the number of most relevant features, and the assigned name for the best model

Dataset	ML algorithm	Most relevant features	AUC ROC score	Accuracy score	Precision score	Recall score	F1 score	Model name
Antimalarial-strain 3D7	Random Forest	40	0.69	0.82	0.58	0.33	0.42	MLSyPred©-Mal3D7
Antimalarial-strain Dd2	Logistic Regression	44	0.81	0.79	0.45	0.76	0.59	MLSyPred©-MalDd2
Antimalarial-strain HB3	Logistic Regression	30	0.70	0.68	0.32	0.64	0.43	MLSyPred©-MalHB3

Evaluation metrics include AUC ROC, precision, recall, and F1 scores. All of these models correspond to the 2048-Morgan fingerprint features

**Table 2:** Synergistic drug combinations obtained in the validation dataset of the antimalarial Dd2 strain using Logistic Regression as the best predictive model

Drug 1	Drug 2	Synergy Prediction
apicidin	dihydroergotamine	Yes
apicidin	hydroxyzine	Yes
apicidin	virginiamycin S1	Yes
dihydroergotamine	trifluoperazine	Yes
guanethidine	trifluoperazine	Yes
hydroxyzine	dihydroergotamine	Yes
sorafenib	hydroxyzine	Yes
sorafenib	trifluoperazine	Yes
virginiamycin S1	dihydroergotamine	Yes

These predictions were determined by a binary classification of Synergy (Yes/No) using 2048-Morgan fingerprint features. The shaded grey row represents the synergistic drug combination confirmed by Kalantar-Motamendi *et al*. [41]

**Table 3:** The best predictive model for the antibiotic dataset, including the ML algorithm, the number of most relevant characteristics, the AUC ROC score, and the assigned name for the best predictive model corresponding to the 166-MACCS key feature fingerprints

Dataset	ML algorithm	Most Relevant Features	AUC ROC score	Model name
Antibiotics	Random Forest	48	0.88	MLSyPred©-Antb

## Data Availability

The supplemental material and code used to build the machine learning models are freely available on GitHub (https://github.com/rcmi-igpd/MLSyPred). The source code is free and open to all users. The MLSyPred© tool and the source code are copyrighted under registration number TXu 002-263-404.

## Abbreviations

ACTs: artemisinin combination therapies
ML: Machine learning
MLSyPred©: Machine learning synergy predictor
*Ci*: Correlation index
RACS: Ranking-system of anti-cancer synergy
DIGRE: Drug-induced genomic residual effect
CoSynE: Combinations synergy estimation
ETL: Extract, transform, load
IDE: Integrated development environment
SMILES: Simplified molecular input line entry system
MACCS: Molecular access system
RF: Random Forests
LR: Logistic Regression
SVM: Support vector machine
GB: Gradient boost
AB: AdaBoost
CV: Cross-validation
SOMTE: Synthetic minority over-sampling technique
RUS: Random undersampling
RFE: Recursive Feature Evaluation
AUC ROC: Area under the curve receiver operating characteristic
TPR: True Positive Rate
FPR: False Positive Rate
MLSyPred©-Mal3D7: Machine learning synergy predictor’s best model for *P. falciparum* strain 3D7
MLSyPred©-MalDd2: Machine learning synergy predictor’s best model for *P. falciparum* strain Dd2
MLSyPred©-MalHB3: Machine learning synergy predictor’s best model for *P. falciparum* strain HB3
MLSyPred©-Antb: Machine learning synergy predictor’s best model for *E. coli* antibiotics
